# Victims of drug facilitated sexual assault aged 13-24: a cross sectional study on the pool of users of a sexual violence relief centre in Northern Italy

**DOI:** 10.1007/s00414-024-03197-0

**Published:** 2024-02-20

**Authors:** Cinzia Simonaggio, Elena Rubini, Giulia Facci, Paola Castagna, Antonella Canavese, Lorenza Scotti, Sarah Gino

**Affiliations:** 1 School of Medicine, University of Turin, Corso Dogliotti, 38, Torino, 10126 Italy; 2 CRIMEDIM - Center for Research and Training in Disaster Medicine, Humanitarian Aid and Global Health, University of Eastern Piedmont, Novara, 28100 Italy; 3 Centro Soccorso Violenza Sessuale, Presidio Ospedaliero Sant’Anna, Città della Salute e della Scienza, corso Spezia, Torino, 60 – 10126 Italy; 4 Department of Surgical Sciences, University of Turin, corso Dogliotti 14, Torino, 10126 Italy; 5 Department of Translational Medicine, University of Eastern Piedmont, Via Solaroli 17, Novara, 28100 Italy; 6 Department of Health Sciences, University of Eastern Piedmont, via Solaroli 17, Novara, 28100 Italy

**Keywords:** Substance use, Adolescents, Gender-based violence (GBV), Sexual violence, Drug facilitated sexual assault (DFSA), Forensic toxicology

## Abstract

This cross-sectional study aimed to assess the association between drugs and alcohol intake and sexual abuse in adolescents, otherwise defined as Drug Facilitated Sexual Assault (DFSA). We considered the survivors who accessed care at the Centre “Soccorso Violenza Sessuale” (SVS – Sexual Violence Relief Centre) in Turin (Italy), between May 2003 and May 2022. We found that 973 patients aged 13–24 among which 228 were victims of DFSA. Epidemiological and anamnestic aspects of the episode of sexual violence were examined, with a specific focus on investigating the alcohol and/or drug intake as reported by the victim, along with the results of the toxicological analysis. the study further accounts for the variations caused by the COVID-19 pandemic on DFSA-related accesses. Our findings show that 23% of adolescents accessing care at SVS were subjected to DFSA. Six out ten adolescents knew their aggressor, at times a partner (10%) oran acquaintance (43%). In 12% of cases violence was perpetrated by a group of people (12%). Almost 90% of young victims described alcohol consumption, while 37% reported drug use at the time of the assault. Alcohol taken alone or in combination with other substances was the most detected drug in our sample throughout the period considered. Given the large use of psychoactive substances among adolescents, it is imperative to implement harm reduction strategies alongside educational activities aimed at fostering awareness about consent. Health personnel should be trained to manage the needs of victims of DFSA clinically and forensically.

**Supplementary Information:**

The online version contains supplementary material available at 10.1007/s00414-024-03197-0.

## Background

Adolescents tend to engage in risky behaviours more frequently than adults [1–3], with higher rates of alcohol or other psychoactive substances consumption [1, 4–6]. Reports show that 61% of seniors students in the United States of America (US) drink alcohol [7], as well as 18% of Italians aged between 11 and 17 and 73% of those aged 18–24 who consumed alcohol at least once in their lives, in 66% of cases describing consumption as “occasional” [8]. Furthermore, 26% of young Italians (15–19 of age) have tried one or more psychoactive drug including marijuana, cocaine, stimulants, hallucinogens, and opiates, with marijuana being the most consumed illicit substance [9]. In Italy, one in four students uses cannabis, followed by new psychoactive substances, synthetic cannabinoids, and stimulants [8]. In the US, 44% of college students [10], 33% of 16-year-old and 15% of 14-year-old students, have used it at least once in their lifetime [7].

One in three women has experienced some form of violence (sexual, physical, or both) during her lifetime, and younger women are also at risk [11]. Rates of sexual violence victimization are high among transgender people and adolescents [12, 13]Sexual violence in adolescents is widespread, and affects half of the population between 15 and 24 years of age in some geographical areas [11].

The state of unconsciousness deriving from self-administered drugs (alcohol and other substances) can be exploited by perpetrators to carry out various forms of violence, including sexual abuse, and is defined as opportunistic drug facilitated sexual assault (DFSA) [15, 16] Conversely, proactive DFSA occurs when drugs are intentionally used by perpetrators or their accomplices to compromise a person’s ability to consent to sexual activity [15, 17] a situation that is increasingly adversely impacting adolescents and young women [16]. In this case, substance consumption can be forced, or the victim could be unaware of the presence of drugs. Having any kind of sexual interaction with a person who is unable to fully consent is a crime and constitutes sexual violence. Some sources estimate that 75% of all forms of violence concern the consumption of alcohol or drugs [17], whereas a study conducted in Spain in 2011 estimates DFSA cases as 31% of the overall sexual violence occurrences [18]. In Italy a study conducted at Brescia Hospital reported that 22% of all victims of sexual offences were subjected to DFSA [19]. Alcohol is one of the psychoactive substances most frequently involved in rape [17], along with gamma-Hydroxybutyric acid (GHB), ketamine, benzodiazepines (e.g., Rohypnol, Valium, Xanax), analgesics (e.g., Fentanyl, Codeine, Tramadol), and antidepressants (e.g., Citalopram, Fluoxetine, Amitriptilina) [16, 17, 20, 21]. Several studies have documented the association between drug use and sexual violence in adolescents [22–27]. Muchimba defines sexual violence and the use of substance as predicting variables in the general population [21]. Given that young women and transgender people are at increased risk of sexual abuse, that the use of alcohol and marijuana is more frequent in adolescents, and keeping into consideration that some of these substances and their effects are used or exploited by perpetrators of DFSA, analysing the intersection of these elements could serve to better understand the phenomenon of DFSA in adolescents.

To our knowledge, no systematic studies focusing on adolescents and DFSA were conducted in Italy. Therefore, the aim of this study is to describe the characteristics of the pool of users aged 13–24 accessing care at the Centre “Soccorso Violenza Sessuale” (SVS – Sexual Violence Relief Centre) in Turin, North-West Italy, who were victims of DFSA, and their epidemiology in terms of demographic characteristics, relationship with perpetrators, as well as physical and psychological outcomes, evaluating the relationship between these variables and alcohol or drug consumption events in the context of DFSA. Furthermore, the study accounts for the impact of Covid-19 on the reduced number of accesses to the SVS Centre. The study was guided by the questions: “What are the demographic characteristic, the relationship with perpetrators, and alcohol or drug consumption habits of patients accessing care at SVS in cases of DFSA?” and “How did the Covid-19 pandemic impact DFSA trends in the study population?”

## Materials and methods

### Study design

We conducted a retrospective cross-sectional study, evaluating the medical records of all survivors assisted by the Centre “Soccorso Violenza Sessuale” (SVS - Sexual Violence Relief Centre)^1^ of Sant’Anna Hospital, an Obstetrics and Gynaecology facility in Turin, Italy, from May 2003 to May 2022. To protect patients’ privacy, the medical records are in paper form and kept in locked file cabinets in a location accessible only by SVS staff.

This cross-sectional study analysed the history of assault of patients aged 13–24 who reported being subjected to sexual abuse and declared alcohol or drug intake or had physical or manifested psychological signs or symptoms of alcohol or drug use, and for whom a complete medical record accompanied by the report of toxicological investigations carried out at a second level regional laboratory^2^ was available. For the purpose of the present study, the term “adolescent” will be used interchangeably with “patient”, “survivor”, and “victim” to refer to our study population, namely individuals between 13 and 24 years of age.

Medical records were eligible for inclusion when they related to cases of DFAS in adolescents as per the operational definition. Conversely, when they related to patients in other age groups, or who were not victims of DFSA they were excluded. No time filter was applied to the screening, as all medical records collected since the opening of SVS until the start of the study were eligible.

The study was approved by the Ethical Committee of the “A.O.U. Città della Salute e della Scienza di Torino – A.O. Ordine Mauriziano di Torino” (CE 112/2020) and was organised according to the Declaration of Helsinki for experiments involving humans (2013), to the General Data Protection Regulation (2018) and to the Provision no. 146/2019 of the Italian Privacy Guarantor.

### Data collection

In Online Resource 1 was reported the information that was extrapolated from the medical records by one of the authors (C.S.) and checked by one of the centre’s health professionals (A.C.)

### Statistical analysis

Descriptive statistics were calculated to summarize the information collected, specifically, categorical variables were reported as absolute frequencies and percentages. Variables with a frequency lower or equal to five were aggregated for the subsequent analyses. The Chi square or Fisher exact tests were used to evaluate the association between subjects’ characteristics and drugs and alcohol consumption. Moreover, univariable Poisson regression models with robust variance were applied to estimate the prevalence ratios (PRs) and corresponding 95% confidence intervals for the association between harassment characteristics and alcohol or drugs consumption. The time trend in the prevalence of alcohol and drugs use was also assessed and chi square test used to evaluate the difference between years, only information regarding the latest years (2018–2022) were used for this analysis to focus on most recent trends. The statistical significance level was set at 0.05. Statistical analyses were performed using SAS Version 9.4 (SAS Institute, Cary, North Carolina).

## Results

Table 1 in Online Resource 2 contains the descriptive statistics of the categorical variables discussed below, including demographics and episode-specific information, while Table 2 in Online Resource 3 summarizes the distribution of survivors’ demographic characteristics, violence, physical and psychological outcomes and their relationship with alcohol and drug intake, the p-value of the test used to assess the association as well as the prevalence ratios (PRs) and corresponding 95% confidence intervals (95% CI).

### Population enrolled

**Fig. 1:**
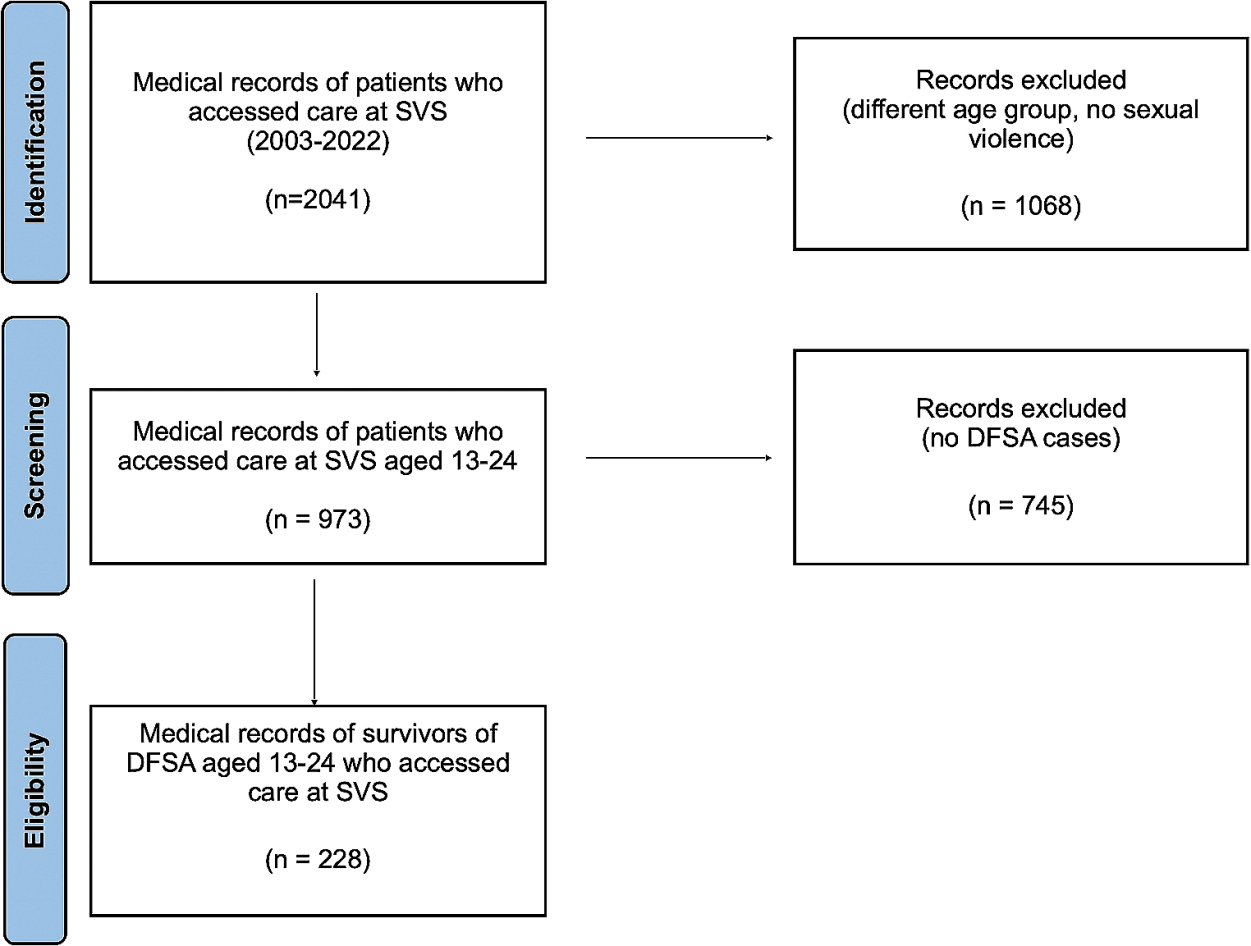
Screening of sources

Since 2003, 2041 survivors accessed care at SVS, an average of 102.05 (± 37.03) per year. 973 (48%) of the patients were between 13 and 24 years of age, an average of48.7 (± 19.6) subjected to sexual violence per year. The final sample consisted of 228 (23%) adolescent survivors of DFSA with an average of 11.5 (± 7.2) per year. Figure 1 shows the screening of sources.

### Demographic characteristics

In our sample, 22% of victims were in the age group 13–16, 34% were aged 17–19, and 44% between 20 and 24 years old. Most victims were cisgender women, and one was a transgender man. In 227 of the 228 sexual assaults (99%) aggressors were male and only in one case the perpetrator was a woman. As for the geographical origin, 69% of the survivors were Italian, 11% came from Eastern Europe (e.g., Albania and Romania), 3% from other European countries, 2% from Africa, and 15% had other geographical origins.

In most cases (82%), sexual assaults fell within the conditions of official reporting in the Italian Criminal Code, while only 62% of the victims declared that they wanted to file a complaint.

### Violence findings

During their interview with gynaecologist and midwife, survivors reported details about the assault, including the place where violence occurred, although in 14 cases the victim was unable to describe or remember what happened. Sexual violence could be perpetrated in leisure places such as bars, pubs, clubs, restaurants (16%), public places (22%), or other locations (10%), however 50% of all the violence took place at home. Only 1% of sexual assaults occurred in a working environment.

The time elapsed between the episode of sexual violence and the arrival at the SVS was analysed: survivors accessed care more frequently during the first 12 h after the event (41%), in the first 6 h (18%) and between 6 and 12 h (23%). A reduction in access follows (19% between 12 and 24 h and 13% in the 24–48 h), up to a new peak after 48 h from the event (27%).

The relationship between victim and aggressor was also evaluated: 60% of survivors knew their aggressor (10% a partner, 43% an acquaintance, 12% a group of acquaintances, which for the purpose of this study could also include the victim’s partner). Only in 22% of cases violence had been perpetrated by a stranger, alone or in a group (12%), and in 25 cases the young woman was unable to describe her aggressor.

### Psychological and physical findings

A frequent psychological symptom was amnesia, reported alone in 12% of cases and in association with other symptoms in 44% of survivors. The psychological impact of rape on young patients was significant: 30% experienced feelings of anger, anxiety, and shame, as well as fear of possible future consequences. One in ten adolescents (10%) experienced emotional dissociation (e.g., feelings of detachment or estrangement from others) [29] following sexual assault. Only 1% and 0.45% of women experienced emotional numbness and hallucinations.

Among the 168 adolescents who complained of adverse physical outcomes, abdominal-pelvic pain was the most reported (49%).

While 46% of survivors had no injuries at the time of the medical examination, when present, lesions were caused by blunt instruments (44%) and bladed weapons (1%), often used as threat tools. The most affected body area was face and neck (18%). Multiple lesions targeting several body parts (30%), at time also involving genital area of victims were found (15%).

### Alcohol and drugs intake

Regarding alcohol consumption 89% of patients reported having drunk alcohol, and 11% denied consumption. In 84% of cases, adolescents described voluntarily having consumed alcohol, while 3% disclosed they were forced under threats of beatings or reprisals, and 2% reported they ingested it unknowingly. Adolescents described having drunk hard liquors (76%), beer (13%), and wine (11%). In some cases (*n* = 21), victims were not able to recognize the type of drink or did not explicitly report it. Among the 202 patients who had taken alcohol, 39% drank it in leisure places (e.g., pubs, bars, restaurants), while 32% of adolescents preferred private homes. Only 19% consumed alcoholic beverages during parties or raves, and 10% referred they drank alcohol in unconventional venues (e.g., parking lots, parks, or cars). Seven victims were unaware of their location at the time of consuming alcohol or were unable to describe it.

The use and presence of other drugs was also analysed. One in three girls (37%) reported they had voluntarily used drugs, while 48 women denied, but based on their subsequent symptoms they could not exclude that drug consumption had occurred without their knowledge.

Within the sample of 228 patients, in 77% of cases a toxicological test was carried out. Among the biological matrices analysed, the most used were the combination of blood and urine (71%). In 50% of cases, corresponding to 113 female patients, the drugs used during the violence were detected. Among the 113 adolescents, alcohol was the most frequently detected substance with a positive result in 57% of cases, alone (32%) or in combination, followed by cannabinoids (30%), alone (5%) or in combination, sedatives (25%), anaesthetics (19%), including ketamine and cocaine, antidepressants (11%), antipsychotics (6%), opioids (3%), and other drugs (12%). This data refers to an absolute positivity of the toxicological examination for the individual categories analysed, which could be connected to a single intake as well as to polysubstance use. In our sample, 45 out of 113 patients (39%) have experimented 27 different combinations of drugs, with the most frequent being cannabinoids together with alcohol (6%).

The association between alcohol consumption and the place where the sexual violence occurred, as well as with the presence of physical symptoms were analysed. Specifically, alcohol consumption was more likely in sexual assaults occurring in leisure places (51%) compared to sexual abuse perpetrated in private homes (18%). When sexual violence led to physical consequences, victims were less likely to have consumed alcohol (PR 0.90. 95%CI 0.83–0.97).

Women over the age of 16 are less likely to use drug compared to adolescents aged 13–16 (43% in age group 17–19, 36% in age group 20–24). When there was a group of perpetrators, the victim was more likely to have consumed drugs. When perpetrators of gang rape were strangers, drug consumption increased 2.11 times, and when members of the group were survivors’ acquaintances, presence of substances was 2.70 times higher. An inverse relationship was found between drug use, with subjects who consumed alcohol being less likely to take other substances (24%) (PR 0.4, 95% CI 0.29–0.57). No difference was observed in the likelihood of drug use between different types of alcohol, while in cases of sexual abuse that occurred in leisure places it was less likely that the victim had used other drugs compared to abuses that occurred in private homes.

**Fig. 2:**
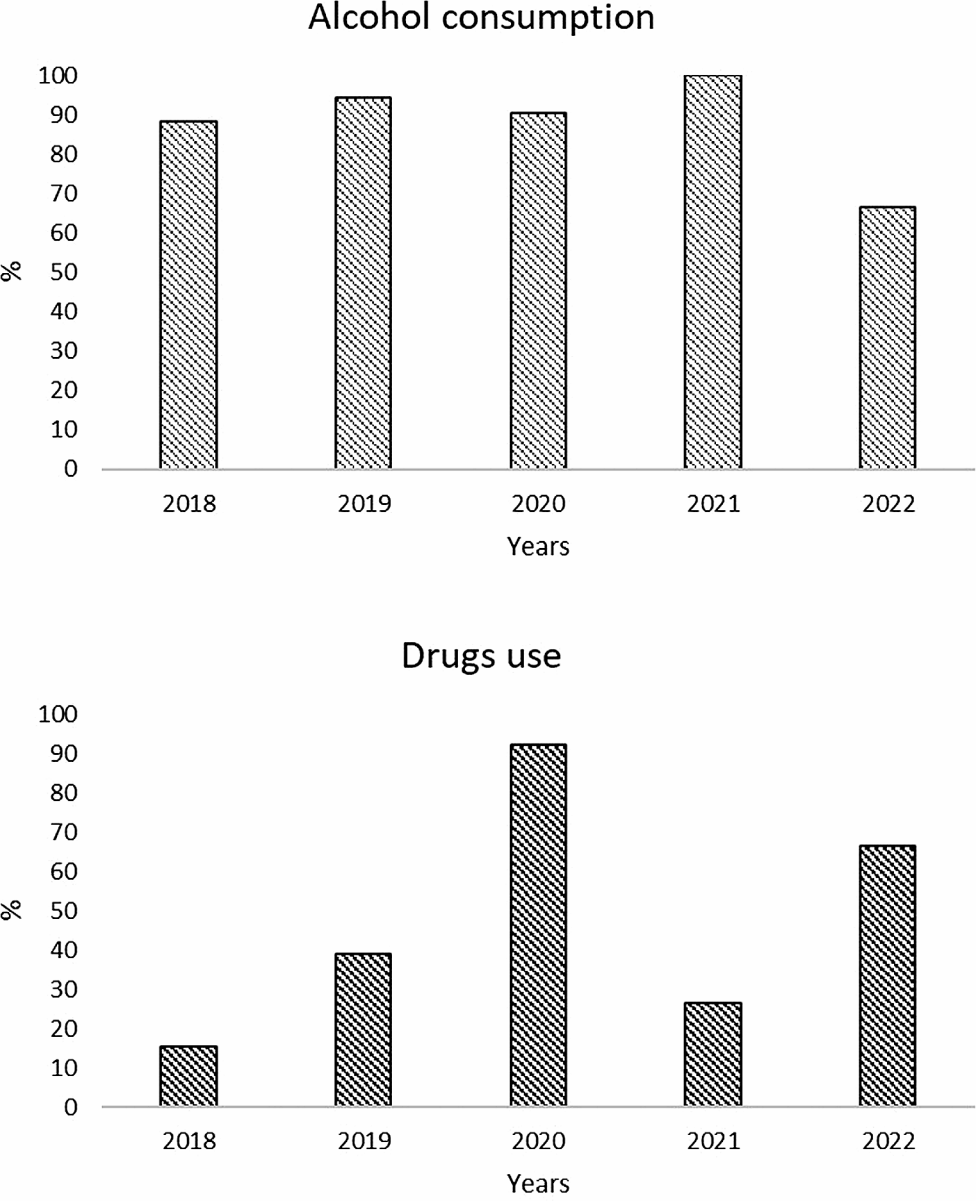
Trend of alcohol and other drugs intake between 2018 and 2022 among abused adolescents

Among patients, alcohol consumption ranged from 67% in 2020 to 100% in 2021, with no statistically significant difference observed between years (p-value 0.2980), while the frequency of drug use increased from 2018 (15%) to 2020 (92%) with a decrease in 2021 (27%) (Fig. 2).

### Pandemic results

Additionally, the study aimed to explore the impact of the Covid-19 pandemic on the reported episodes of DFSA in adolescents. To understand the distribution pattern over the years of the pandemic, the data are presented disaggregated by year for the whole period. Moreover, the period spanning from 2020 to 2022 was further segmented into four distinct phased, aligning with the two periods of lockdown in Italy and the subsequent easing of lockdown restrictions,. The number of adolescents examined at SVS in this timeframe was 38 (17% of the overall study sample). During the first lockdown phase (between March 9, 2020 and May 4, 2020) two patients accessed the centre. During the second phase, the post-lockdown during the summer of 2020, 14 patients accessed the SVS. Four survivors accessed during the third phase under examination, corresponding to the second lockdown (between November 3, 2020 and April 26, 2021). During the months following the end of the second lockdown 18 victims accessed the service (from April 26, 2021 to May 2022).

As far as the use of drugs is concerned, considering the entire pandemic period, only three adolescents reported not having consumed alcohol and in 88% of the cases the intake was voluntary.

In the same three-year period, there was a reduction in the consumption of alcoholic beverages in leisure places, due to the restrictions and regulations put in place during the pandemic. Households became the location where most violence occurred (45%). Furthermore, due to the restrictive measures, young people moved their leisure activities to public parks, parking lots, campers, and warehouses (18%). This also had an impact on drug consumption: in particular, 58% patients used drugs (including in 31% of cases of possible unaware consumption of substances), while 42% denied it. Overall, in 2020, 92% of adolescents accessing care at SVS reported having used alcohol and other drugs.

Similarly, all toxicological tests performed during lockdowns were positive; in particular, during the third phase under investigation, corresponding to the second lockdown, all tests resulted positive for alcohol, alone or in combination.

## Discussion

The study allows an assessment of the epidemiological characteristics of adolescent victims of DFSA, from which it can be deduced that this population is the most represented age group accessing care at SVS (48%). This result is in line with the findings highlighted by the “WHO Multi-country Study on Women’s Health and Domestic Violence against Women” [30], based on data from nine countries collected between 2000 and 2004, but is lower compared to data collected by the Italian Institute of Statistics (ISTAT) in a survey on violence against women conducted in Italy in 2014 [31]. In our study, 23%, of adolescents accessing care at SVS were subjected to DFSA, the majority being Italians (67%). This is in contrast with ISTAT findings, which reported women of other geographical origins (20%, against 13% of Italian women) as the most affected by physical or sexual violence, perpetrated by their current or former partner [36]. In our study, although 22% of the abuses were perpetrated by a stranger, in 43% of cases the perpetrator was an acquaintance, a fellow student, a friend, or a partner (10%). At times, assault involved multiple strangers (12%), friends, or acquaintances, often together with the survivor’s partner (12%).

The majority of adolescents (69%) sought health care within 24 h after violence occurred, while alarmingly 27% delayed their arrival at hospital beyond 48 h.

In 46% of cases injuries were not described.

When physical sequelae were reported, survivors complained of abdominal and pelvic pain, headache, and nausea, alone or in combination. One in six adolescents reported full or partial amnesia, either alone or in combination with other symptoms. These findings are in line with other studies where a positive association between DFSA and symptoms of nausea and amnesia was found [32]. However, when the victim experienced drowsiness and confusion, on awakening the prevailing feelings were anger, fear, or shame for what had happened (30%) up to emotional detachment. Amnesia induced by alcohol and drugs was so impactful that for the survivor it was not possible to remember or clearly define the place where the violence occurred (*n* = 14) or who the perpetrator was (*n* = 25). Fields and colleagues focused precisely on the psychological impact of DFSA, and on amnesia being a possible cause of Post-Traumatic Stress Disorder [33]. Conversely, when sexual violence led to the appearance of physical symptoms, victims were less likely to have consumed alcohol (PR 0.90. 95%CI 0.83–0.97). This can be partially explained by drink-induced amnesia: an aggressor could more easily abuse unconscious victims, unable to defend themselves, and thus cause fewer injuries and symptoms.

The study also underlines the variety of substances that can be used by perpetrators to sexually violate a victim: alcohol plays an undisputed role (in nine out of ten sexual assaults). The association of alcohol with DFSA, especially among students, has already been confirmed by several authors [23, 26, 34]. Parker also found that adolescents who regularly use alcohol and marijuana are at higher risk of experiencing verbal or physical violence [35]. Adolescents voluntary consumed alcohol in 84% of cases, a situation that can be fully associated with opportunistic assault; only in fewer instances adolescents were forced to drink or were unaware of the presence of alcohol in their drinks. Furthermore, alcohol-related violence occurs more often in private homes (51.32%) than in leisure places, such as pub or discos (17.99%).

Survivors reported voluntarily consumption of other drugs in 37% of cases, while 48 adolescents only disclosed subsequent symptoms. Curiously, in our study there is an inverse relationship between alcohol and drug intake: women who used drugs were 24% less likely to consume alcohol. Furthermore, the age group that most reported psychoactive substances consumption was the youngest (13–16 years old). In a study conducted by Mognetti, women belonging to younger age groups and those aged over 50 denied voluntary consumption of substances, while in our study the younger group (13–16 years) was the first in terms of drug use [36].

Our study confirmed how drugs are used in contexts of social aggregation or in the private domestic sphere, with recreational purposes [37] that are exploited in opportunistic sexual assault by aggressors, mostly partners or friends. In a study conducted by Prego-Meleiro many rapes, occurring in the context of nightlife, fell within the scope of opportunistic assault and mostly concerned young women [38]. A study conducted in Australia highlighted the association of voluntary drug use with DFSA [39] and another project in Norway underlined the difficulties in finding evidence of proactive sexual assault [40]. The voluntary intake of alcohol and other substances in DFSA cases was also confirmed by a study previously conducted at the SVS Centre, focusing on all DFSA cases, regardless of victims’ age, from 2008 to 2017 [36]. Although covering a shorter period compared to the present study, the voluntary assumption of alcohol (87%) and drugs (43%) was frequent. A second study, conducted in the same centre, involved a comparison between survivors who were subjected to DFSA and a control group [41]. Interestingly, its findings show how DFSA was more prevalent when violence occurred in public places or in houses different from the one of the victims, and women aged 18–25 were considered the group at higher risk of victimization [41]. In a study conducted in Denmark [42] 70% of all victims of DFSA between 2019 and 2020 were between the age of 15 and 25 and during 2020 62% of the overall victims of DFSA had voluntarily consumed alcohol, a figure lower than our results.

Additionally, we saw that violence perpetrated by a group was more likely to be associated with drug use. This result is in line with a study conducted in Brescia (Italy), in which 52% of cases of group-perpetrated sexual violence were associated with drugs intake compared to 19% substance use in cases of violence perpetrated by a single individual [19].

For 113 patients the toxicological investigation gave a positive result, even if not always in line with patients’ declarations. Furthermore, the spread of new and hard-to-detect drugs, and an excessive time interval between sexual violence and medical examination represent a major challenge [16, 17, 21].

Sexuality education is needed, to prevent gender-based violence, including DFSA, and to raise awareness in the younger generations on consent [43]( Any sexual act performed on a person without their full consent, given without coercion or use of force, constitutes a crime and a violation of their human rights, including their sexual and reproductive rights, no matter if the state of unconsciousness or dizziness was caused by voluntary consumption of substances. In Italy, efforts should be made to include opportunistic DFSA as an aggravating factor for perpetrators of sexual abuse, adapting to regulations already put into practice in other States [44]. At the same time, health personnel managing cases of sexual assault should be trained to be able to take care of the needs of victims from a clinical and forensic perspective in cases of opportunistic as well as of proactive DFSA.

Alcohol was the most frequently identified psychoactive substance alone (57.5%) and together with cannabinoids (30.1%), sedatives (24.8%), and anaesthetics (19.5%). adhering to findings in the scientific literature in Italy [45] and in other countries [16, 17, 20, 32, 40, 46]. Given the large use of psychoactive substances among adolescents implementation of harm reduction strategies is needed.

The SARS-COV2 pandemic has exacerbated this situation with increase in cases of adolescents referred to SVS between 2020 and 2022, representing more than half of all victims of sexual abuse. Conversely, in the literature a decrease in adolescent access to care during times of social confinement or lockdown was observed, with an increase in the number of cases with the easing of restrictions [47].

Even though the limited sample prevents us from statistically analyse data, the descriptive findings show how alcohol consumption among adolescents has not decreased during the pandemic. Conversely, almost half of the cases related to the use of this psychoactive substance occurred at home, exceeding the average of the total period considered in our study (32%) voluntary.

As established by several studies, substance use remained constant [48] or increased [49] during the lockdown. In our sample, 58% of the patients involved in our study declared that they had used drugs in the overall period and in 2020 92% of adolescents admitted at SVS had taken psychoactive substances.

Our study has some limitations. First, we analysed a population of adolescents who had experienced DFSA, without considering a control group, so differences among our study population and victims of non-DFSA could not be analysed. Moreover, due to our operational definition, we could not properly describe the differences between cases of DFSA when substances were self-administered and not. More studies are needed comparing outcomes and prevalence of opportunistic and proactive DFSA. Furthermore, although the study covers a very large period of 19 years, the limited sample restricts the statistical capabilities for analysing the phenomenon As for DFSA cases occurring during the Covid pandemic the extremely limited sample size does not allow robust statistical analysis. Finally, our study might not have fully captured the outcomes and the incidence of drugs employed by perpetrators when they could not be detected through toxicological exams.

## Conclusion

Our study outlines the epidemiology of DFSA cases among adolescents accessing care at SVS in Turin. In this facility, adolescents were the most represented age group. In cases of DFSA opportunistic assaults were much more frequent compared to unaware consumption and proactive assaults. In this context, voluntary consumption was often exploited by acquaintances or partners, especially in private homes. The age group 13–16 was the one with the higher prevalence of self-administered alcohol and other substances use. Sexuality education encompassing notions of consent is needed. Policymakers in Italy should include opportunistic DFSA as an aggravating factor for perpetrators of sexual violence, aligning to regulations put in place in other States. Implementation of harm reduction strategies is also imperative.

### Electronic supplementary material

Below is the link to the electronic supplementary material.

Supplementary Material 1

Supplementary Material 2

Supplementary Material 3

## Footnotes

1 SVS is one of the two Italian sexual violence relief centres open 7 days a week, 24 h per day and represents the reference centre in the metropolitan area of Turin (North-West Italy), which has about 2,250,000 inhabitants. A multidisciplinary team (gynaecologists, midwives, psychologists, social workers, and medical examiners) and takes care of sexually abused women. as well as of mistreated pregnant women and migrant women who have suffered any form of violence.

2 The laboratory is accredited UNI CEI EN ISO/IEC 17025:2018 and performs toxicological exams as reported in the Gruppo Tossicologi Forensi Italiani (Italian Association of Forensic Toxicologists) Guidelines for toxicological examinations due to forensic and other medico-legal reasons (“Linee guida per la determinazione di sostanze stupefacenti e psicotrope su campioni biologici con finalità tossicologico-forensi e medico-legali”) [28].
