# Supplementary material for: Victims of drug facilitated sexual assault aged 13-24: a cross sectional study on the pool of users of a sexual violence relief centre in Northern Italy
Source: Int J Legal Med. 2024 Feb 20;138(4):1593–602. doi: 10.1007/s00414-024-03197-0 (PMC11164715; doi:10.1007/s00414-024-03197-0)
Supplement: Supplementary file 1 — Supplementary Material 1 [file 414_2024_3197_MOESM1_ESM.docx]

**Data collected from medical records**

- *Anamnestic data*: when sexual violence occurred, the date of arrival at the SVS, the age of the victim, divided into 3 age groups (13-16, 17-19, and 20-24 years); the victim’s geographical origin (Italian, Eastern European, European, African, and Other countries).
- *Violence data*: the place where the violence occurred (public location, private homes, defined as the place where the victim, the perpetrator, or other acquaintances lived, workplaces, pubs, bars, discos, cars, campsites, parking lot, fields, warehouses, or multiple places); the time elapsed between the violence and the admission to the Centre (0-6 hours, 6-12 hours, 12-24 hours, 24-48 hours or > 48 hours); number of abusers (one or more); the pre-existing relationship between the aggressor and the victim (unknown, acquaintance, partner, boyfriend, or a friend).
- *Clinical Data*: physical symptoms (pain, nausea, weakness/fatigue, dizziness, vomiting/diarrhoea, headache, or the combination of several symptoms) and psychological manifestations (numbness, amnesia, hallucinations, emotional detachment, anger, fear, anxiety, shame, guilt, or a combination of multiple symptoms with or without amnesia); the type of lesion (blunt force injury, sharp force injury, , or the combination of multiple injuries) or presence of scar outcomes; the area of the body involved (head and neck, trunk, upper limbs, lower limbs, genitals and other erogenous sites, or a combination, including the genitals or not).
- *Data on drug use*: considerable attention was paid to psychoactive substances, evaluating victims’ stories to distinguish between cases of voluntarily, forced, or unaware consumption; the place where victims took drugs (pubs, bars, restaurants, homes, parties, raves, or other places). Alcohol was considered separately from other substances and differentiated between drinking beer, wine, and hard liquor. Data obtained from the history of violence was integrated with the results of the toxicological analysis, conducted on different biological matrices (e.g., blood, urine, hair, or different combinations). The drugs detected were divided into nine main categories, based on their Anatomical Therapeutic Chemical (ATC) classification: anaesthetics, opioids, cannabinoids, sedatives, antipsychotics, antidepressants, drugs acting on the CNS (e.g., buprenorphine), alcohol, or belonging to other categories (e.g., analgesics).
- *Data on reports sent to the Judicial Authority* by healthcare professionals according to the Italian Criminal Code and whether the victim wanted to file a complaint or not.
