# Supplementary material for: Victims of drug facilitated sexual assault aged 13-24: a cross sectional study on the pool of users of a sexual violence relief centre in Northern Italy
Source: Int J Legal Med. 2024 Feb 20;138(4):1593–602. doi: 10.1007/s00414-024-03197-0 (PMC11164715; doi:10.1007/s00414-024-03197-0)
Supplement: Supplementary file 2 — Supplementary Material 2 [file 414_2024_3197_MOESM2_ESM.docx]

**Table 1**- Distribution of the demographical characteristics of the victims of sexual abuse and characteristics related to the episodes.

|  | **N=228** |
| --- | --- |
| **Age range** | **N (%)** |
| 13-16 years | 50 (22) |
| 17-19 years | 77 (34) |
| 20-24 years | 101 (44) |
| **Place** |  |
| Private homes | 107 (50) |
| Others (cars, camping, parking, fields, warehouses) | 21 (10) |
| Workplace | 3 (1) |
| Leisure places (pubs, bars, discos) | 35 (16) |
| Public place | 48 (22) |
| Missing | 14 |
| **Victim’s nationality** |  |
| African | 4 (2) |
| Others | 35 (15) |
| East Europe | 25 (11) |
| European | 7 (3) |
| Italian | 157 (69) |
| **Time elapsed between violence and medical examination** |  |
| 0-6 hours | 41 (18) |
| 12-24 hours | 44 (19) |
| 24-48 hours | 29 (13) |
| 6-12 hours | 51 (23) |
| >48 ore hours | 60 (27) |
| Missing | 3 |
| **Aggressor** |  |
| Acquaintance | 88 (43) |
| Groups of strangers | 24 (12) |
| Groups of acquaintances | 25 (12) |
| Partner | 21 (10) |
| Unknown | 45 (22) |
| Missing | 25 |
| **Physical symptoms** |  |
| Abdominal and pelvic pain | 83 (49) |
| Headache | 32 (19) |
| Weakness/fatigue | 7 (4) |
| Nausea | 19 (11) |
| Combination of symptoms | 25 (15) |
| Vomiting/diarrhoea | 2 (1) |
| Missing | 60 |
| **Mechanism of injury** |  |
| None | 106 (46) |
| Bladed weapons | 2 (1) |
| Blunt instruments | 100 (44) |
| Bladed weapons and blunt instruments | 4 (2) |
| Blunt instruments | 1 (1) |
| Scar outcomes | 15 (6) |
| **Site of the injury** |  |
| Lower limbs | 15 (12) |
| Upper limbs | 10 (8) |
| Head and neck | 22 (18) |
| Trunk | 5 (4) |
| Genitals and other erogenous sites | 14 (11) |
| Different locations (genitals included) | 19 (15) |
| Different locations (genitals excluded) | 37 (30) |
| **Psychological symptoms** |  |
| Hallucinations | 1 (0.45) |
| Amnesia | 26 (12) |
| Emotional detachment | 22 (10) |
| Numbness | 3 (1) |
| Multiple symptoms (amnesia included) | 98 (44) |
| Multiple symptoms (amnesia excluded) | 6 (3) |
| Others (anger, fear, anxiety, shame, guilt) | 66 (30) |
| **Official report** |  |
| No | 41 (18) |
| Yes | 187 (82) |
| **Complaint** |  |
| No | 72 (37) |
| Yes | 120 (62) |
| Missing | 36 |
| **Declared alcohol consumption** |  |
| No | 24 (11) |
| Voluntary | 187 (83) |
| Unintentional | 4 (2) |
| Forced | 8 (3) |
| **Type of alcoholic beverage** |  |
| Beer | 23 (13) |
| Hard liqueur | 135 (76) |
| Wine | 20 (11) |
| Missing | 21 |
| **Place of alcohol consumption** |  |
| Private homes | 61 (32) |
| Leisure places (pubs, bars, discos) | 76 (39) |
| Party, rave | 36 (19) |
| Others | 19 (10) |
| Missing | 7 |
| **Declared drugs consumption** |  |
| No | 114 (63) |
| Yes | 66 (37) |
| Missing | 48 |
| **Toxicological test** |  |
| No | 52 (23) |
| Yes | 176 (77) |
| **Biological matrix** |  |
| Hair | 9 (5) |
| Blood | 11 (6) |
| Blood and urine | 126 (71) |
| Blood, urine, and hair | 3 (2) |
| Urine | 23 (13) |
| Urine and hair | 4 (2) |
| **Substance** |  |
| Others | 8 (7) |
| Others/alcohol | 1 (1) |
| Others/anaesthetics/opioids/sedatives/antidepressants | 1 (1) |
| Others/anaesthetics /cannabinoids | 1 (1) |
| Others/cannabinoids | 1 (1) |
| Others/cannabinoids/alcohol | 1 (1) |
| Others/Alcohol | 1 (1) |
| Anaesthetics | 8 (7) |
| Anaesthetics/cannabinoids | 3 (3) |
| Anaesthetics/cannabinoids/sedatives /alcohol | 1 (1) |
| Anaesthetics/cannabinoids/antidepressants/alcohol | 1 (1) |
| Anaesthetics/cannabinoids/alcohol | 4 (3) |
| Anaesthetics/sedatives/alcohol | 1 (1) |
| Anaesthetics/antidepressants/alcohol | 1 (1) |
| Anaesthetics/alcohol | 1 (1) |
| Opioids/sedatives/antipsychotics | 1 (1) |
| Opioids/sedatives/antipsychotics/antidepressants | 1 (1) |
| Opioids/alcohol | 1 (1) |
| Cannabinoids | 6 (5) |
| Cannabinoids/sedatives | 3 (3) |
| Cannabinoids/sedatives/antipsychotics | 2 (1) |
| Cannabinoids/sedatives/antipsychotics/antidepressants/alcohol | 1 (1) |
| Cannabinoids/sedatives/antipsychotics/Alcohol | 1 (1) |
| Cannabinoids/sedatives /antidepressants | 1 (1) |
| Cannabinoids/sedatives/alcohol | 1 (1) |
| Cannabinoids/alcohol | 7 (6) |
| Sedatives | 8 (7) |
| Sedatives/antidepressants | 2 (2) |
| Sedatives/antidepressants/alcohol | 1 (1) |
| Sedatives/alcohol | 3 (3) |
| Antipsychotics | 1 (1) |
| Antidepressants | 1 (1) |
| Antidepressants/alcohol | 2 (2) |
| Alcohol | 36 (32) |
| Missing | 63 |
| **Number of adolescents who accessed care at SVS per year** |  |
| 2003 | 6 (3) |
| 2004 | 4 (2) |
| 2005 | 4 (2) |
| 2006 | 3 (1) |
| 2007 | 7 (3) |
| 2008 | 10 (4) |
| 2009 | 5 (2) |
| 2010 | 17 (7) |
| 2011 | 13 (6) |
| 2012 | 4 (2) |
| 2013 | 7 (3) |
| 2014 | 14 (6) |
| 2015 | 12 (5) |
| 2016 | 15 (7) |
| 2017 | 18 (8) |
| 2018 | 26 (11) |
| 2019 | 18 (8) |
| 2020 | 23 (10) |
| 2021 | 19 (8) |
| 2022 | 3 (1) |
| **Number of adolescents who accessed care at SVS during the pandemic (2020 – 2022)** |  |
| 9 March 2020 - 4 May 2020 (first lockdown period) | 2 (1) |
| 5 May 2020-2 November 2020 | 14 (6) |
| 3 November 2020 - 26 April 2021(second lockdown period) | 4 (2) |
| > 26 April 2021 | 18 (8) |
