# Supplementary material for: Victims of drug facilitated sexual assault aged 13-24: a cross sectional study on the pool of users of a sexual violence relief centre in Northern Italy
Source: Int J Legal Med. 2024 Feb 20;138(4):1593–602. doi: 10.1007/s00414-024-03197-0 (PMC11164715; doi:10.1007/s00414-024-03197-0)
Supplement: Supplementary file 3 — Supplementary Material 3 [file 414_2024_3197_MOESM3_ESM.docx]

**Table 2** - Distribution of women’s demographic characteristics, violence, physical and psychological results according to alcohol and drug use, the p-value of the test used to assess the association as well as the prevalence ratios (PRs) and corresponding 95% confidence intervals (95%CI).

|  | **Alcohol use** | | | | **Drugs use** | | | |
| --- | --- | --- | --- | --- | --- | --- | --- | --- |
|  | **No N=24** | **Yes N=199** | **p-value** | **PR (95%CI)** | **No N=114** | **Yes N=66** | **p-value** | **PR (95%CI)** |
|  | **N (%)** | **N (%)** |  |  | **N (%)** | **N (%)** |  |  |
| **Age range** |  |  |  |  |  |  |  |  |
| 13-16 years | 8 (33.33) | 41 (20.6) | 0.2343 | 1 | 20 (17.54) | 22 (33.33) | 0.0501 | 1 |
| 17-19 years | 5 (20.83) | 70 (35.18) |  | 1.12 (0.97-1.28) | 42 (36.84) | 18 (27.27) |  | 0.57 (0.35-0.93) |
| 20-24 years | 11 (45.83) | 88 (44.22) |  | 1.06 (0.92-1.22) | 52 (45.61) | 26 (39.39) |  | 0.64 (0.42-0.98) |
| **Place** |  |  |  |  |  |  |  |  |
| Private homes | 9 (40.91) | 97 (51.32) | 0.0151* | 1 | 53 (47.75) | 30 (48.39) | 0.2935 | 1 |
| Leisure places (pubs, bars, discos) | 0 (0.00) | 34 (17.99) |  | 1.09 (1.03-1.16) | 22 (19.82) | 6 (9.68) |  | 0.59 (0.28-1.27) |
| Public place (cars, camping, parking, fields, warehouses) | 9 (40.91) | 39 (20.63) |  | 0.89 (0.77-1.03) | 26 (23.42) | 20 (32.26) |  | 1.20 (0.78-1.86) |
| Others/workplace | 4 (18.18) | 19 (10.05) |  | 0.90 (0.74-1.10) | 10 (9.01) | 6 (9.68) |  | 1.04 (0.52-2.08) |
| Missing | 2 | 10 |  |  | 3 | 4 |  |  |
| **Country of origin of the victim** |  |  |  |  |  |  |  |  |
| Italy | 17 (70.83) | 137 (68.84) | 1.0000* | 1 | 82 (71.93) | 49 (74.24) | 0.5776 | 1 |
| Europe | 3 (12.5) | 29 (14.57) |  | 1.019 (0.30-1.15) | 14 (12.28) | 10 (15.15) |  | 1.11 (0.66-1.88) |
| Outside Europe | 4 (16.67) | 33 (16.58) |  | 1.00 (0.89-1.14) | 18 (15.79) | 7 (10.61) |  | 0.75 (0.38-1.46) |
| **Time elapsed between violence and medical examination** |  |  |  |  |  |  |  |  |
| 0-6 hours | 5 (20.83) | 36 (18.18) | 0.9208* | 1 | 20 (17.54) | 14 (21.54) | 0.3224 | 1 |
| 12-24 hours | 6 (25) | 37 (18.69) |  | 0.98 (0.83-1.16) | 21 (18.42) | 15 (23.08) |  | 1.01 (0.58-1.77) |
| 24-48 hours | 3 (12.5) | 26 (13.13) |  | 1.02 (0.86-1.21) | 19 (16.67) | 6 (9.23) |  | 0.58 (0.26-1.30) |
| 6-12 hours | 4 (16.67) | 45 (22.73) |  | 1.05 (0.91-1.21) | 29 (25.44) | 11 (16.92) |  | 0.67 (0.35-1.27) |
| >48 ore hours | 6 (25) | 54 (27.27) |  | 1.03 (0.89-1.18) | 25 (21.93) | 19 (29.23) |  | 1.05 (0.62-1.77) |
| Missing |  | 1 |  |  |  | 1 |  |  |
| **Aggressor** |  |  |  |  |  |  |  |  |
| Unknown | 7 (30.43) | 37 (21.02) | 0.0279* | 1 | 28 (27.18) | 12 (19.67) | <0.0001 | 1 |
| Acquaintance | 4 (17.39) | 82 (46.59) |  | 1.13 (0.99-1.30) | 48 (46.6) | 18 (29.51) |  | 0.91 (0.49-1.68) |
| Partner | 2 (8.7) | 19 (10.8) |  | 1.08 (0.89-1.30) | 16 (15.53) | 2 (3.28) |  | 0.37 (0.09-1.49) |
| Groups of strangers | 4 (17.39) | 19 (10.8) |  | 0.98 (0.78-1.23) | 7 (6.80) | 12 (19.67) |  | 2.11 (1.17-3.78) |
| Groups of acquaintance | 6 (26.09) | 19 (10.8) |  | 0.90 (0.70-1.17) | 4 (3.88) | 17 (27.87) |  | 2.70 (1.61-4.53) |
| Missing | 1 | 23 |  |  | 11 | 5 |  |  |
| **Physical symptoms** |  |  |  |  |  |  |  |  |
| Pain | 14 (63.64) | 66 (46.15) | 0.3331* | 0.98 (0.81-1.20) | 42 (50.00) | 28 (56.00) | 0.8002 | 1.00 (0.54-1.84) |
| Headache | 2 (9.09) | 30 (20.98) |  | 1.12 (0.92-1.35) | 17 (20.24) | 7 (14.00) |  | 0.73 (0.32-1.66) |
| Weakness/fatigue/nausea/vomiting/diarrhoea | 2 (9.09) | 26 (18.18) |  | 1.11 (0.91-1.35) | 13 (15.48) | 7 (14.00) |  | 0.88 (0.39-1.95) |
| Combination of symptoms | 4 (18.18) | 21 (14.69) |  | 1 | 12 (14.29) | 8 (16.00) |  | 1 |
| Missing | 2 | 56 |  |  | 30 | 16 |  |  |
| **Physical symptoms** |  |  |  |  |  |  |  |  |
| No | 2 (8.33) | 56 (28.14) | 0.0367 | 1 | 30 (26.32) | 16 (24.24) | 0.7586 | 1 |
| Yes | 22 (91.67) | 143 (71.86) |  | 0.90 (0.83-0.97) | 84 (73.68) | 50 (75.76) |  | 1.07 (0.68-1.69) |
| **Type of injury** |  |  |  |  |  |  |  |  |
| None | 13 (54.17) | 89 (44.72) | 0.6459 | 1 | 49 (42.98) | 31 (46.97) | 0.8252 | 1 |
| Blunt instruments and Others/Bladed weapons | 10 (41.67) | 96 (48.24) |  | 1.04 (0.94-1.14) | 56 (49.12) | 31 (46.97) |  | 0.92 (0.62-1.36) |
| Outcomes | 1 (4.17) | 14 (7.04) |  | 1.07 (0.92-1.25) | 9 (7.89) | 4 (6.06) |  | 0.79 (0.34-1.88) |
| **Lesions** |  |  |  |  |  |  |  |  |
| No | 13 (54.17) | 89 (44.72) | 0.3804 | 1 | 49 (42.98) | 31 (46.97) | 0.6039 | 1 |
| Yes | 11 (45.83) | 110 (55.28) |  | 1.04 (0.95-1.14) | 65 (57.02) | 35 (53.03) |  | 0.90 (0.62-1.33) |
| **Psychological symptoms** |  |  |  |  |  |  |  |  |
| Other (anger, fear, anxiety, shame, guilt) | 4 (18.18) | 62 (31.96) | 0.0636* | 1 | 36 (32.43) | 18 (28.57) | 0.2167 | 1 |
| Amnesia | 0 (0.00) | 26 (13.4) |  | 1.07 (1.00-1.13) | 16 (14.41) | 4 (6.35) |  | 0.60 (0.23-1.56) |
| Emotional detachment/Numbness | 3 (13.64) | 21 (10.82) |  | 0.93 (0.79-1.10) | 11 (9.91) | 11 (17.46) |  | 1.50 (0.85-2.63) |
| Multiple symptoms with/without amnesia | 15 (68.18) | 85 (43.81) |  | 0.91 (0.82-1.00) | 48 (43.24) | 30 (47.62) |  | 1.15 (0.72-1.85) |
| Missing | 2 | 5 |  |  | 3 | 3 |  |  |
| **Official report** |  |  |  |  |  |  |  |  |
| No | 4 (16.67) | 34 (17.09) | 1.0000* | 1 | 19 (16.67) | 10 (15.15) | 0.7899 | 1 |
| Yes | 20 (83.33) | 165 (82.91) |  | 1.00 (0.88-1.12) | 95 (83.33) | 56 (84.85) |  | 1.08 (0.63-1.85) |
| **Complaint** |  |  |  |  |  |  |  |  |
| No | 7 (31.82) | 64 (38.32) | 0.6440* | 1 | 36 (36.73) | 19 (34.55) | 0.7865 | 1 |
| Yes | 15 (68.18) | 103 (61.68) |  | 0.97 (0.87-1.07) | 62 (63.27) | 36 (65.45) |  | 1.06 (0.68-1.66) |
| Missing | 2 | 32 |  |  | 16 | 11 |  |  |
| **Alcohol consumption** |  | | | |  |  |  |  |
| No |  |  |  |  | 6 (5.31) | 18 (27.69) | <0.0001 | 1 |
| Yes |  |  |  |  | 107 (94.69) | 47 (72.31) |  | 0.41 (0.29-0.57) |
| Missing |  |  |  |  | 1 | 1 |  |  |
| **Type of alcohol** |  |  |  |  |  |  |  |  |
| None |  |  |  |  | 6 (5.41) | 18 (33.33) | <0.0001 | 2.58 (1.76-3.76) |
| Beer |  |  |  |  | 14 (12.61) | 6 (11.11) |  | 1.03 (0.49-2.15) |
| Hard liqueur |  |  |  |  | 73 (65.77) | 30 (55.56) |  | 1 |
| Wine |  |  |  |  | 18 (16.22) | 0 (0.00) |  | NA |
| Missing |  |  |  |  | 3 | 12 |  |  |
| **Place of alcohol consumption** |  |  |  |  |  |  |  |  |
| No |  |  |  |  | 6 (5.45) | 18 (29.03) | <0.0001 | 1.71 (1.16-2.55) |
| Private homes |  |  |  |  | 27 (24.55) | 21 (33.87) |  | 1 |
| Leisure places (pubs, bars, discos) |  |  |  |  | 40 (36.36) | 14 (22.58) |  | 0.59 (0.34-1.03) |
| Party, rave |  |  |  |  | 27 (24.55) | 3 (4.84) |  | 0.23 (0.08-0.70) |
| Others |  |  |  |  | 10 (9.09) | 6 (9.68) |  | 0.86 (0.42-1.74) |
| Missing |  |  |  |  | 4 | 4 |  |  |
| **Drugs consumption** |  |  |  |  |  |  |  |  |
| No | 6 (25) | 107 (69.48) | <0.0001 | 1 |  | | | |
| Yes | 18 (75) | 47 (30.52) |  | 0.76 (0.65-0.89) |  |  |  |  |
| Missing |  | 45 |  |  |  |  |  |  |
| **Toxicological test** |  |  |  |  |  |  |  |  |
| No | 3 (12.5) | 49 (24.62) | 0.1846 | 1 | 32 (28.07) | 12 (18.18) | 0.1369 | 1 |
| Yes | 21 (87.5) | 150 (75.38) |  | 0.93 (0.85-1.02) | 82 (71.93) | 54 (81.82) |  | 1.46 (0.86-2.46) |
